# Prolactin Role in COVID-19 and Its Association with the Underlying Inflammatory Response

**DOI:** 10.3390/ijms252211905

**Published:** 2024-11-06

**Authors:** Eleni Polyzou, Georgios Schinas, Panagiotis Bountouris, Dimitra Georgakopoulou, Anne-Lise de Lastic, Anastasia Parthymou, Charalambos Gogos, Venetsana Kyriazopoulou, Athanasia Mouzaki, Anastasia Theodoropoulou, Karolina Akinosoglou

**Affiliations:** 1 School of Medicine, University of Patras, 26504 Rio, Greece; polyzou.el@gmail.com (E.P.); georg.schinas@gmail.com (G.S.); delastic@gmail.com (A.-L.d.L.); cgogos@upatras.gr (C.G.); vkyriazopoulou@med.upatras.gr (V.K.); mouzaki@upatras.gr (A.M.); 2 Department of Internal Medicine, University General Hospital of Patras, 26504 Rio, Greece; 3 Division of Endocrinology, Department of Internal Medicine, University General Hospital of Patras, 26504 Rio, Greece; panosbountouris@yahoo.com (P.B.); theodorop@upatras.gr (A.T.); 4 Immunology Laboratory, 251 Hellenic Air Force General Hospital, 11525 Athens, Greece; dimgeorg9@gmail.com (D.G.); natassap@yahoo.com (A.P.); 5 Division of Infectious Diseases, Department of Internal Medicine, University General Hospital, 26504 Patras, Greece

**Keywords:** COVID-19, prolactin, biomarkers, disease severity, cytokines, immunomodulation, inflammatory markers, sex differences, cohort studies, viral infections

## Abstract

The COVID-19 pandemic has prompted interest in identifying reliable biomarkers to predict disease severity and guide clinical decisions. Prolactin (PRL), a hormone traditionally associated with lactation, has gained attention for its role in immune modulation. This study aimed to assess PRL as a biomarker for disease severity in COVID-19. A prospective cohort of 142 patients with moderate to severe COVID-19, defined as a WHO-CPS 5 or 6, was recruited from the University General Hospital of Patras. Baseline PRL levels were measured using an electrochemiluminescence immunoassay, and serum cytokines, including IL-1β, IL-6, IL-8, IL-10, IL-12p70, and TNF-α, were quantified through flow cytometry. Clinical outcomes, including mortality and the need for invasive mechanical ventilation (IMV), were recorded. Results indicated that PRL levels were significantly higher in female patients (12.95 ng/mL vs. 9.40 ng/mL, *p* < 0.001) but they did not correlate with key severity indices such as CCI, SOFA score upon admission or inflammatory markers. No significant associations between baseline PRL levels, cytokine concentrations, and clinical outcomes in COVID-19 were noted. Our findings suggest that PRL may lack prognostic reliability for disease severity compared to more established predictive markers and that its role in the immune response remains uncertain.

## 1. Introduction

The COVID-19 pandemic has challenged global healthcare systems, driving extensive research into biomarkers for disease severity and progression. Given the complexity of the immune response in COVID-19, reliable biomarkers obtained early in the course of the disease could potentially guide and optimize patient management during hospitalization [1]. COVID-19 hospitalization has been recognized as a severe event that significantly impacts quality of life and the functional status of the respiratory system [2]. While many studies have focused on ambitious or novel predictors [3], we adopted a different approach by investigating a basic hormone, prolactin (PRL).

PRL, a hormone traditionally associated with lactation and reproduction, has emerged as a promising candidate due to its multifaceted role in the immune system [4]. Prolactin is known to stimulate both innate and adaptive immunity in humans, with its effects mediated through endocrine, paracrine, and autocrine mechanisms [5,6,7,8]. Recent studies have highlighted the potential of PRL as an immunomodulator, capable of eliciting both pro-inflammatory and immunosuppressive responses depending on its concentration and the physiological context. [6,9,10,11,12,13]

Prolactin’s influence on the immune system is complex and far-reaching. It acts as a cytokine, signaling through pathways similar to those used by other immune mediators. In adaptive immunity, PRL promotes the proliferation and differentiation of T-lymphocytes, enhances B-cell antibody production [5,6], and modulates the activity of natural killer cells. In the innate immune response, PRL stimulates phagocytosis and the production of reactive oxygen species in macrophages and neutrophils [6,14]. Importantly, PRL has been shown to regulate the expression of cytokines such as IL-1β, IL-6, and TNF-α, which are key players in the inflammatory response observed in severe COVID-19 cases. The hormone’s ability to shift the balance between Th1 and Th2 responses further highlights its potential significance in modulating the immune response to SARS-CoV-2 infection. [15,16].

The role of prolactin in viral infections has been priorly explored and seems to go hand in hand. Hyperprolactinemia occurs in 21.4% of people living with HIV and is related to higher CD4+ cell counts [17]. The human cytomegalovirus enhances the expression of prolactin receptors (PRLRs) in ovarian cancer by activating inflammatory signaling pathways and inhibiting anti-inflammatory mechanisms, which not only facilitate viral replication but also intensify the inflammatory response, possibly sharing common immunological pathways [18]. PRLRs could serve as a receptor and entry site for virus-host cell communication for other viruses as well [19]. Increased PRL serum levels have been observed in patients with HCV and RSV [20,21]. During the 2003 SARS pandemic, SARS-CoV caused a significant rise in serum PRL levels due to dysregulation of the adeno-pituitary control [22]. This imbalance was attributed to either the virus’s direct cytopathic effects or the accompanying pro-inflammatory changes

The gender disparity observed in COVID-19 outcomes, where men face higher risks of severe disease and mortality regardless of age, has led researchers to investigate potential hormonal influences in different outcomes [23]. Notably, women generally exhibit higher baseline PRL levels than men, a difference that persists beyond menopause [24]. This observation aligns with the hypothesis that PRL may play a protective role in COVID-19 [4]. Furthermore, the unexpected lower infection rates among psychiatric patients treated with medications known to increase PRL levels, such as chlorpromazine, tricyclic antidepressants, and SSRIs [25], have strengthened the case for investigating PRL’s potential protective effects against SARS-CoV-2 infection [26,27].

This study aims to investigate the role of PRL as a biomarker for disease severity in COVID-19 and its potential modulatory effects on the immune response in moderate to severe cases, filling a gap in the current literature, as no prior studies have systematically examined prolactin’s role in the complex immunological mechanisms of COVID-19 or its prognostic utility. By evaluating baseline PRL levels and their correlation with key inflammatory cytokines, we seek to elucidate PRL’s potential utility in guiding clinical decisions and improving patient outcomes.

## 2. Results

### 2.1. Population Characteristics

The demographic and clinical characteristics of the study population are summarized in Table 1. The median age of the patients was 70 years (IQR: 56–81), with 58 (40.8%) being female. Prolactin (PRL) levels had a median concentration of 10.20 ng/mL (IQR: 8.10–16.15). PRL concentrations were significantly higher in female patients compared to males, with a median concentration of 12.95 ng/mL (IQR: 9.00–20.30) in females and 9.40 ng/mL (IQR: 7.60–13.55) in males (*p* < 0.001). PRL concentrations did not correlate with age or severity indices (SOFA, CCI), nor were they significantly different across survival or invasive mechanical ventilation (IMV) or vaccination subgroups.

### 2.2. Subgroup Analysis

The demographic and clinical characteristics of the study population are presented in Table 2 and Table 3. Table 2 compares survivors and non-survivors, showing that non-survivors were older (median age 73 vs. 68 years, *p* = 0.0367), had higher SOFA scores (median 3 vs. 2, *p* = 0.0001), a higher Charlson Comorbidity Index (CCI) (median 4 vs. 3, *p* = 0.0027), and a longer length of hospital stay (median 15 vs. 8 days, *p* < 0.0001). There were no significant differences in prolactin levels between these two subgroups.

Table 3 presents a comparison between male and female patients. Females were significantly older (median age 74 vs. 68 years, *p* = 0.0385) and had a shorter length of hospital stay (median 7 vs. 10 days, *p* = 0.0079). Other variables, including SOFA score and CCI, showed no significant differences between sexes. A correlation analysis was conducted between baseline prolactin (PRL) levels and all inflammatory markers stratified by sex. Among all the inflammatory markers, only Ferritin was significantly correlated with PRL levels in the female subgroups. (Spearman’s Rho = 0.327, *p* = 0.017).

### 2.3. Study of Inflammatory Response

The baseline characteristics of the subset of the population sampled-at-baseline are detailed in Table 4. The inflammatory profile and immune response characteristics of the subgroup, including interleukin values at the baseline, are also summarized. The median age of this subgroup was 69 years (IQR: 57–82), with 26 (33.8%) being female. The median prolactin level was 10.40 ng/mL (IQR: 8.55–16.10). PRL levels were not significantly different across vaccination subgroups (*p* = 0.355). PRL concentrations did not correlate with age, CCI, or inflammatory markers, nor were they significantly different across survival subgroups or IMV subgroups. Finally, PRL measurements did not correlate with interleukin levels at the baseline.

### 2.4. ROC Curve Analysis

**Figure 1:**
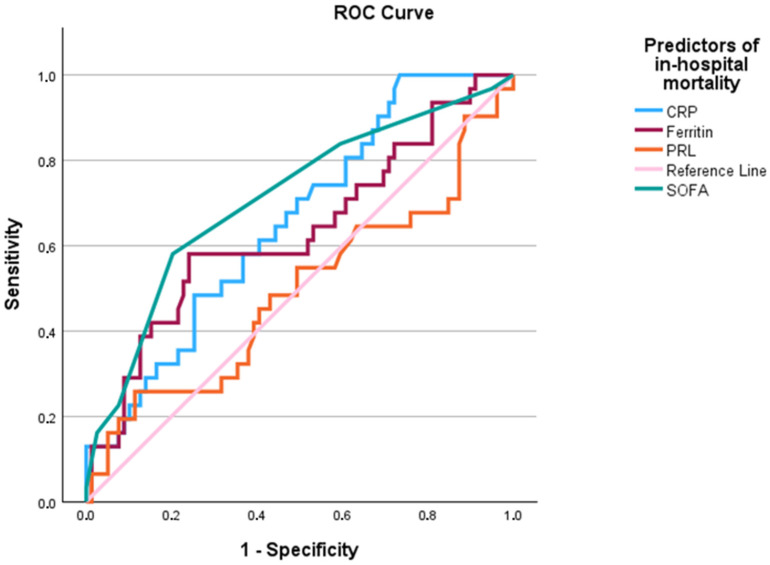
ROC curve analysis for the outcome of in-hospital mortality. Abbreviations: CRP; C-Reactive Protein, PRL; Prolactin, SOFA; Sequential Organ Failure Assessment score.

**Figure 2:**
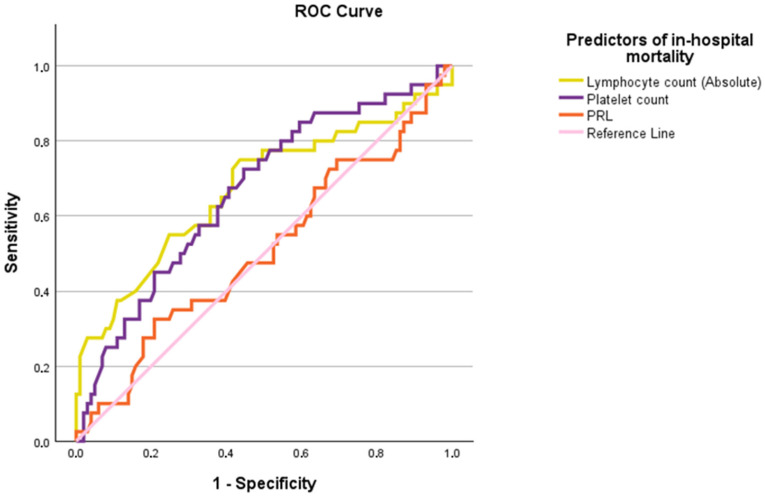
ROC curve analysis for the outcome of in-hospital mortality. Abbreviations: PRL; Prolactin.

The ability of various inflammatory and disease severity markers to predict in-hospital mortality in COVID-19 patients was assessed using the Receiver Operating Characteristic (ROC) curve analysis. The Area Under the Curve (AUC) for each marker is presented in Figure 1 and Figure 2. Among the markers analyzed, SOFA had the highest predictive value for in-hospital mortality, with an AUC of 0.712, indicating moderate predictive potential. CRP (AUC = 0.655) and ferritin (AUC = 0.632) also demonstrated moderate predictive capabilities. In this context, PRL’s AUC was less than 0.5. Absolute lymphocyte count had the highest predictive value among the inverse predictors, with an AUC of 0.673, followed closely by platelet count with an AUC of 0.666, indicating moderate predictive ability when values were low. PRL showed minimal predictive potential with an AUC of 0.508, reinforcing its limited role in predicting in-hospital mortality when values are low.

## 3. Discussion

This was the first study to systematically investigate prolactin’s role in COVID-19, specifically aiming to assess its potential as a biomarker. We examined whether prolactin concentrations correlate with disease severity or worse outcomes and evaluated correlations with key interleukins involved in COVID-19 inflammatory mechanisms to support its role in immunological pathways, and our findings provide valuable insights into the role of PRL in COVID-19 patients with moderate to severe disease. Contrary to our initial hypothesis, PRL levels did not demonstrate significant elevations or correlations with most clinical outcomes or inflammatory markers. However, several noteworthy observations emerged.

To begin with, we did not find increased (or decreased) levels of PRL in COVID-19 patients and no correlation with most clinical outcomes or inflammatory markers that could allow for its use in diagnosis or prognosis of the disease. Previous studies have suggested elevated PRL in COVID-19 cases compared to non-infected patients; however, study heterogeneity is noted, leading authors to conclude that infection-related stress and not COVID-19 per se is possibly what primarily drives hyperprolactinemia [4,28,29,30]. Of note, despite the absence of specific genotypic analysis in our study, a significant portion of our cohort data originates from the period dominated by the Omicron variant, which is associated with less severe disease, in contrast to previous studies performed during the alpha or delta mutant era [31,32]. It is possible that this accounts for the discrepancy of PRL levels between studies, since different mutants are prone to diverse immunologic responses [33,34]. In the same context, a proportion of our population was previously vaccinated and/or immune, eliciting different responses [35].

Secondly, the expected gender difference in PRL levels was confirmed, with female patients exhibiting significantly higher concentrations than males. Prolactin levels vary throughout women’s lives. They are higher in premenopausal women than in postmenopausal women. After menopause, prolactin levels do not differ between men and women [36,37]. Several studies suggest that gender differences impact vulnerability, severity, and outcomes of SARS-CoV-2 infection, with men prone to developing severe COVID-19 and facing a higher risk of mortality compared to women [38]. However, differing prolactin levels between men and women may only partly account for the observed variations in disease progression. Even though there is evidence that prolactin exhibits immunomodulatory properties [39], data have shown that hormonal shifts, immune responses, and lifestyle habits may be responsible for these differences, as well as other sex hormones [40,41]. Notably, in a cohort of climacteric women with moderate COVID-19, prolactin levels were significantly elevated during the acute phase of infection, alongside reductions in estradiol, testosterone, and other key hormones; however, at 12 months post-infection, prolactin levels had significantly decreased in 73.3% of these patients compared to their acute phase levels (*p* = 0.041) [42]. In the current study, the increased levels of prolactin observed in women compared to men could perhaps explain a faster recovery in women, as evidenced by shorter hospitalization times (median 7 vs. 10 days, *p* = 0.007), despite similar parameters of severe disease.

Last, as previously established, patients of older age, co-morbidities and more severe disease, as this is reflected in increased CCI and SOFA scores have increased risk of mortality [43,44]. These patients present with marked lymphopenia or thrombopenia, increased inflammatory indices and acute phase proteins including CRP, ferritin and d-Dimers. Several of those markers, similar to our study have been identified as prognostic indices, with significant diagnostic capacity [45,46,47]. While traditional markers like SOFA, CCI, CRP, Ferritin, and D-dimers provide moderate prognostic value, PRL does not contribute significantly to mortality in this cohort.

It should be noted that elevated PRL levels have been associated with improved outcomes in sepsis due to them enhancing immune function, suggesting a potential benefit in critical illnesses [48,49]. Olmos-Ortiz et al., showed that PRL reduces LPS-induced inflammation by decreasing TLR4 and NF-κB expression, leading to lower levels of pro-inflammatory cytokines like TNF-α, IL-1β, and IL-6 [50]. On the other hand, in another study of septic patients, elevated serum PRL levels were linked to acute lung injury [51]. PRL plays a complex role in the immune response, exhibiting both pro-inflammatory and anti-inflammatory effects [4,52]. Studies have suggested elevated PRL in COVID-19 cases compared to non-infected patients; however, study heterogeneity is noted [28,29]. Similarly to various viral diseases, such as HIV, HCV and CMV infection, the increased PRL levels are attributed to PRL’s role in viral entry and replication, as well as the induction of inflammatory-signaling pathways [4]. In COVID-19 high PRL levels might reflect an attempt by the body to counterbalance the excessive inflammation and support immune defense [4]. In this setting, IL-6 remains a critical cytokine in the inflammatory response in COVID-19 disease. It is reported that serum IL-6 levels are notably higher in severe cases of COVID-19, and these elevated IL-6 levels are strongly linked to worse clinical outcomes [53]. In cases of stress, such as burns injuries, it seems there is a strong positive relationship between serum PRL levels and the levels of other cytokines such as IL-10, IL-6, and IL-8 [54]. The lack of a significant baseline correlation between PRL and IL-6 could indicate that PRL’s role in the immune response is more nuanced and potentially operates through multiple and indirect pathways.

However, elevated PRL levels might also serve as a compensatory mechanism due to PRL’s anti-inflammatory properties [4,17,18,55]. As proposed, prolactin enhances the immune response by promoting macrophage cytotoxicity, ROS production, and cytokine release, stimulating NK cells and T cells, inhibiting Treg function, and boosting B cell antibody production [56]. Thus, the persistent elevation of PRL can alter B cell function and promote auto-reactivity, contributing to a heightened cellular and humoral immune response. By influencing both T and B cells and stimulating the monocyte–macrophage axis, PRL can induce significant inflammatory changes. This complex interplay suggests that PRL may modulate the immune response in COVID-19 in a manner that is not directly captured by static measurements of IL-6 but which becomes apparent through dynamic changes over time and in the context of disease severity. Conversely, increased levels of IL-6 are often correlated with elevated prolactin levels [57,58], as IL-6-induced activation of the JAK/STAT signaling pathway can stimulate prolactin secretion in response to inflammation.

This study has several limitations that should be considered when interpreting the results. Firstly, the relatively small sample size may have limited our ability to detect statistically significant associations between prolactin levels and clinical outcomes in COVID-19 patients, particularly across different patient subgroups. Additionally, this was a single-center study, which may reduce the generalizability of the findings to broader populations with varying demographics, healthcare practices, or COVID-19 disease profiles. Moreover, the study excluded patients receiving medications that influence prolactin levels, such as certain antidepressants, potentially overlooking relevant data from these populations. The timing of sample collection, although standardized, may not fully capture the dynamic fluctuations of prolactin and cytokine levels during the course of infection, thus limiting the ability to draw conclusions regarding temporal changes. Prolactin can exert both pro and anti-inflammatory effects, depending on the stage of COVID-19 disease [52]. Last, our cohort also included patients irrespective of whether they had thyroid or adrenal associated diseases. Secondary regulation of anterior pituitary PRL [59] release cannot be controlled for in these cases, while PRL can either cause or attenuate inflammation depending on the underlying medical condition [52].

## 4. Materials and Methods

### 4.1. Study Design and Participants

This prospective cohort study was conducted between 2022 and 2023 in the COVID-19 units of the Internal Medicine Department of the University General Hospital of Patras, Greece, and recruited adult patients (age > 18 years) admitted to our hospital with moderate to severe COVID-19, defined as a score of 5 or 6, according to the WHO Clinical Progression Scale (WHO-CPS). Patients who met the inclusion criteria were consecutively recruited and received the standard of care for COVID-19, i.e., Remdesivir, Supplementary Oxygen, Dexamethasone, and anticoagulation. A representative proportion of the patient population was chosen for further inspection of their systemic immune response and their serum was sampled for quantification of cytokines (IL-1β, IL-6, IL-8, IL-10, IL-12p70, TNF-α).

### 4.2. Inclusion and Exclusion Criteria

Patients eligible for the study included those with a confirmed SARS-CoV-2 infection by RT-PCR, exhibiting evidence of ongoing lower respiratory tract infection on imaging (chest X-ray or CT scan), and having a negative urine pregnancy test for women of reproductive age. Patients were excluded if they were pregnant or breastfeeding, on antidepressant therapy (SSRIs, MAOIs, SNRIs), had a known history of dysregulation of the hypothalamo-hypophyseal axis (such as hypophyseal insufficiency, adenomas, or apoplexy) or evidence of it in first testing, or were taking medications that affect PRL levels (including methyldopa, cimetidine, phenothiazines, and barbiturates) or who had priorly received immunomodulatory medication including baricitinib, tocilizumab, or anakinra.

### 4.3. Data and Sample Collection

Patient data collected included demographic information, clinical data, treatment details, laboratory results, including all relevant and available inflammatory markers, and finally disease outcomes, including hospitalization outcome and eventual need for invasive mechanical ventilation (IMV). Blood samples were collected within the first 48 h of hospitalization to measure baseline PRL levels. The timing for blood sample collection was 2–3 h after the patient woke each morning, as this period is known to provide the most accurate PRL concentration readings.

### 4.4. Prolactin and Cytokine Measurement

Serum PRL concentrations were assessed using the electrochemiluminescence immunoassay (ECLIA) technique on the Cobas 6000 E601 analyzer (Roche Diagnostics GmbH, Mannheim, Germany). The Elecsys Prolactin II Kit (Roche, CH, Ref. No. 03203093 190) was employed in accordance with the instructions provided by the manufacturer. This assay is calibrated to the 3rd International Reference Preparation (IRP) WHO Standard 84/500 [60]. Systemic inflammatory response was assessed by determining serum cytokine levels (IL-1β, IL-6, IL-8, IL-10, IL-12p70, TNF-α) using flow cytometry. Cytokine concentrations were measured with a BD FACS Array Bioanalyzer, employing a cytometric bead array (CBA) assay (Human Inflammatory Cytokines Kit, cat#551811, BD Biosciences, Bedford, MA, USA). The assay sensitivity was 7.2 pg/mL for IL-1β, 2.5 pg/mL for IL-6, 3.6 pg/mL for IL-8, 3.3 pg/mL for IL-10, 1.9 pg/mL for IL-12p70, and 3.7 pg/mL for TNF-α, with a measurement range of 20–5000 pg/mL.

### 4.5. Statistical Analysis

The Shapiro–Wilk test was used to test the normality of continuous variables. Continuous variables were compared using the Mann–Whitney U test. Associations between categorical variables were assessed using Pearson’s chi-squared test. When data did not have the appropriate structure for Pearson’s chi-squared test, Fisher’s exact test was used. Correlation between baseline PRL levels and cytokine concentrations was analyzed using Spearman’s correlation coefficient. All tests were two-tailed, with a significance level set at *p* = 0.05. Data analyses were performed using SPSS v. 29.0 (IBM Corp, Armonk, NY, USA).

### 4.6. Ethics

Ethical approval was obtained from the Institutional Review Board (IRB) of the hospital (approval protocol number: 250/07.06.2022). Informed consent was obtained from all participants.

## 5. Conclusions

In conclusion, while this study did not find significant associations between baseline prolactin levels and key clinical outcomes or inflammatory markers in COVID-19 patients, it remains a pioneering effort in evaluating prolactin as a potential biomarker for disease severity. Despite the lack of definitive results, this study is the first to systematically investigate the role of prolactin in the context of COVID-19, as suggested by the previous literature. These findings emphasize the complex nature of the immune response in COVID-19 and point to the need for more research to truly understand how prolactin and other hormones might influence the inflammatory response and disease progression.

## Figures and Tables

**Table 1:** Population Characteristics (*n* = 142).

*Demographics*	
Sex (Female) *n*(%)	58 (40.8%)
Age (years) Median (IQR)	70 (56–81)
Fully Vaccinated *n* (%)	53 (37.3%)
Charlson Comorbidity Index (CCI) Median (IQR)	4 (2–5)
Sequential Organ Failure Assessment (SOFA) Median (IQR)	2 (1–3)
*Outcomes*	
Death *n* (%)	40 (28.2%)
Need for Invasive Mechanical Ventilation (IMV) *n* (%)	24 (16.9%)
Length of Hospital Stay (days) Median (IQR)	9 (5–15)
*Laboratory Values*	
Prolactin (ng/mL) Median (IQR)	10.20 (8.10–16.15)
White Blood Cell Count (WBC, cells/uL) Median (IQR)	6725 (4340–9103)
Neutrophil Count (cells/uL) Median (IQR)	5110 (3215–7310)
Lymphocyte Count (cells/uL) Median (IQR)	760 (500–1250)
Platelet Count (PLT, cells/uL) Median (IQR)	225 (168–287)
C-reactive Protein (CRP, mg/L) Median (IQR)	4.26 (1.49–8.73)
Ferritin (ng/mL) Median (IQR)	480 (227–1092)
D-dimers (mg/L) Median (IQR)	0.80 (0.44–1.63)
Fibrinogen (mg/dL) Median (IQR)	526 (432–636)

Abbreviations: *n*: number; IQR: Interquartile Range.

**Table 2:** Population characteristics depending on mortality.

Variable	Survivors (*n* = 102)	Non-Survivors (*n* = 40)	*p*-Value
*Demographics*			
Sex (Female) *n*(%)	46 (45.1)	12 (30)	
Age (years) Median (IQR)	68 (55–80)	73 (65–85)	0.0367
Fully Vaccinated *n* (%)	36 (35.3)	17 (42.5)	
Sequential Organ Failure Assessment (SOFA) Median (IQR)	2 (1–2)	3 (2–3)	0.0001
Charlson Comorbidity Index (CCI) Median (IQR)	3 (1–4)	4 (3–6)	0.0027
*Outcomes*			
Need for Invasive Mechanical Ventilation (IMV) *n* (%)	2 (2)	22 (55)	
Length of Hospital Stay (days) Median (IQR)	8 (5–12)	15 (9–19)	<0.0001
*Laboratory Values*			
Prolactin (ng/mL) Median (IQR)	10.2 (8.47–16.15)	10.55 (7.05–18.75)	0.8950
White Blood Cell Count (WBC, cells/uL) Median (IQR)	6670 (4180–9178)	6775 (4608–8733)	0.9729
Neutrophil Count (cells/uL) Median (IQR)	4990 (3150–7300)	5600 (3445–7183)	0.6294
Lymphocyte Count (cells/uL) Median (IQR)	880 (540–1270)	525 (368–800)	0.0014
Platelet Count (PLT, cells/uL) Median (IQR)	240 (189–302)	196 (146–236)	0.0024
C-reactive Protein (CRP, mg/L) Median (IQR)	3.17 (1.23–7.94)	5.90 (2.44–10.20)	0.0136
Ferritin (ng/mL) Median (IQR)	437.00 (214.50–884.50)	943.00 (336.00–1649.00)	0.0164
D-dimers (mg/L) Median (IQR)	0.71 (0.41–1.08)	1.52 (0.63–2.12)	0.0009
Fibrinogen (mg/dL) Median (IQR)	524.50 (439.25–639.75)	537.00 (429–602)	0.5964

Abbreviations: *n*: number; IQR: Interquartile Range.

**Table 3:** Population characteristics depending on sex.

Variable	Female (*n* = 58)	Male (*n* = 84)	*p*-Value
*Demographics*			
Age (years) Median (IQR)	74 (60–85)	68 (56–78)	0.03
Fully Vaccinated *n* (%)	20 (34.5)	33 (39.3)	0.56
Sequential Organ Failure Assessment (SOFA) Median (IQR)	2 (1–3)	2 (1–3)	0.27
Charlson Comorbidity Index (CCI) Median (IQR)	4 (2–5)	3 (1–4)	0.06
*Outcomes*			
Length of Hospital Stay (days) Median (IQR)	7 (5–11 )	10 (6–16 )	0.007
Death *n* (%)	12 (20.7)	28 (33.3)	0.10
Need for Invasive Mechanical Ventilation (IMV) *n* (%)	7 (12.1)	17 (20.2)	0.20
*Laboratory Values*			
Prolactin (ng/mL) Median (IQR)	12.95 (9.18–20.02)	9.40 (7.60–13.32)	0.001
White Blood Cell Count (cells/uL) Median (IQR)	5520 (3670–7995 )	6910 (5120–9240 )	0.032
Neutrophil Count (cells/uL) Median (IQR)	3960 (2705–6400 )	5820 (4155–7470 )	0.005
Lymphocyte Count (cells/uL) Median (IQR)	840 (505–1277.50)	730 (500–1120)	0.283
Platelet Count (×10^3^ cells/uL) Median (IQR)	228 (163–297 )	222.50 (179.75–284.25)	0.914
C-reactive Protein (mg/L) Median (IQR)	3.17 (1.27–7.79)	5.13 (1.97–9)	0.125
Ferritin (ng/mL) Median (IQR)	294 (139–710)	741 (336–1576)	0.0001
D-dimers (mg/L) Median (IQR)	0.73 (0.39–1.04)	0.89 (0.46–1.72)	0.054
Fibrinogen (mg/dL) Median (IQR)	492 (408–590)	559 (451–665)	0.06

Abbreviations: *n*: number; IQR: Interquartile Range.

**Table 4:** Population sampled-at-baseline characteristics (*n* = 77).

*Demographics*	
Sex (Female) *n* (%)	26 (33.8%)
Age (years) Median (IQR)	69 (57–82)
Fully Vaccinated *n* (%)	29 (37.7%)
Sequential Organ Failure Assessment (SOFA) Median (IQR)	2 (1–3)
Charlson Comorbidity Index (CCI) Median (IQR)	4 (2–5)
*Outcomes*	
Death *n* (%)	29 (37.7%)
Intubation *n* (%)	17 (22.1%)
Length of Hospital Stay (days) Median (IQR)	10 (6–16)
*Laboratory Values*	
Prolactin (ng/mL) Median (IQR)	10.40 (8.55–16.10)
White Blood Cell Count (cells/uL) Median (IQR)	6860 (4260–9840)
Neutrophil Count (cells/uL) Median (IQR)	5435 (3308–8080)
Lymphocyte Count (cells/uL) Median (IQR)	760 (500–1257.50)
Red Cell Distribution Width (%) Median (IQR)	13.90 (12.90–15.50)
Hemoglobin (g/dL) Median (IQR)	13.10 (10.85–14.10)
Platelet Count (×10^3^ cells/uL) Median (IQR)	223 (165.50–305.50)
Fibrinogen (mg/dL) Median (IQR)	558 (439–655)
D-dimers (mg/L) Median (IQR)	0.88 (0.46–1.80)
Lactate Dehydrogenase (U/L) Median (IQR)	320 (254–466.75)
C-reactive Protein (mg/L) Median (IQR)	4.69 (1.65–8.76)
Ferritin (ng/mL) Median (IQR)	547 (156–1110)
Troponin I (ng/mL) Median (IQR)	6.75 (5.32–8.75)
*Inflammatory Response*	
Interleukin-1 beta (pg/mL) Median (IQR)	0.67 (0–2.40)
Interleukin-6 (pg/mL) Median (IQR)	26.13 (7.42–76.68)
Interleukin-8 (pg/mL) Median (IQR)	126.10 (65.91–261.10)
Interleukin-10 (pg/mL) Median (IQR)	4.28 (2.15–10.69)
Interleukin-12p70 (pg/mL) Median (IQR)	0 (0–0.69)
Tumor Necrosis Factor-alpha (pg/mL) Median (IQR)	0.72 (0–2.51)

Abbreviations: *n*: number; IQR: Interquartile Range.

## Data Availability

Data can be made available upon reasonable request.
